# Microbial Diversity of Temperate Pine and Native Forest Soils Profiled by 16S rRNA Gene Amplicon Sequencing

**DOI:** 10.1128/MRA.00298-21

**Published:** 2021-05-20

**Authors:** Adenike Eunice Amoo, Olubukola Oluranti Babalola

**Affiliations:** a Food Security and Safety Niche, Faculty of Natural and Agricultural Sciences, North-West University, Mmabatho, South Africa; Georgia Institute of Technology

## Abstract

Most biodiversity measures indicate an ongoing deterioration due to intensifying anthropogenic pressures even though efforts are being intensified worldwide to conserve biodiversity. Knowledge of the implication of land use change on soil bacterial communities is essential for ecosystem restoration. Here, the effect of the conversion of native forests to temperate pine forests on soil bacterial diversity and community composition was investigated. The diversity and composition of the bacterial communities were affected by land use change across the sites.

## Abstract (precis)

Most biodiversity measures indicate an ongoing deterioration due to intensifying anthropogenic pressures even though efforts are being intensified worldwide to conserve biodiversity. Knowledge of the implication of land-use change on soil bacterial communities is essential for ecosystem restoration.

## ANNOUNCEMENT

Soil bacterial communities are integral parts of ecosystem functioning and are highly susceptible to land use change. Native forests, which are significant pools of biodiversity, are being converted to forest plantations to meet the growing needs of agriculture and timber mining of the ever-increasing human population (1–3). The conversion of native forests to plantations alters the diversity and functioning of forest ecosystems. Land use practices that can diminish land degradation garner enormous attention globally (4–6). A keen understanding of the influence of land conversion from native forests to plantations on bacterial communities that control key biogeochemical processes is significant for environmental sustainability.

Using a soil corer, replicate samples (4 soil cores) were collected within multiple tree rows from 2 native forests and 2 plantations located in Tweefontein (−24°58′N, 30.48′E) and Witklip (−25°12′N, 30°56′E), South Africa. Pinus patula is the dominating tree species in the plantations, while Acacia xanthophloea and Celtis africana dominate the native forests (details in references 4 and 7). Soil samples were collected in July 2016 at a 2-cm diameter and 10-cm depth within multiple tree rows (4, 7). Soil samples in plastic bags were transported to the lab on ice and stored at 4°C in the dark until analyzed (DNA extraction was carried out 3 days after sampling). The soil cores were sieved through 2-mm mesh, and genomic DNA was isolated from 0.25 g of soil using the PowerSoil DNA isolation kit (MoBio Laboratories, CA, USA). Examination of bacterial communities was carried out using 16S amplicon sequencing. The 16S rRNA gene V4 variable region was sequenced by the next-generation sequencing service provider Molecular Research LP (Shallowater, TX, USA) using a MiSeq sequencer (Illumina, Inc. San Diego, CA, USA). Using PCR primers 515F and 806R, paired-end reads of 312 bp were obtained. Data were analyzed using Quantitative Insights Into Microbial Ecology (QIIME; v1.9.1) (8). Except where otherwise stated, default parameters were used all through the analysis. The sequences per sample were clustered into operational taxonomic units (OTUs) using SILVA 99 v132 (9). For filtering, mapping, and OTU picking, SortMeRNA was utilized (10).

Total read counts generated were 915,871 with 57,241 average counts per sample. The maximum counts per sample was 178,222, while the minimum was 17,012. After filtering, a total of 21,443 low-abundance features were removed contingent on prevalence. Based on interquantile range, a total of 180 low-variance features were removed.

**FIG 1:**
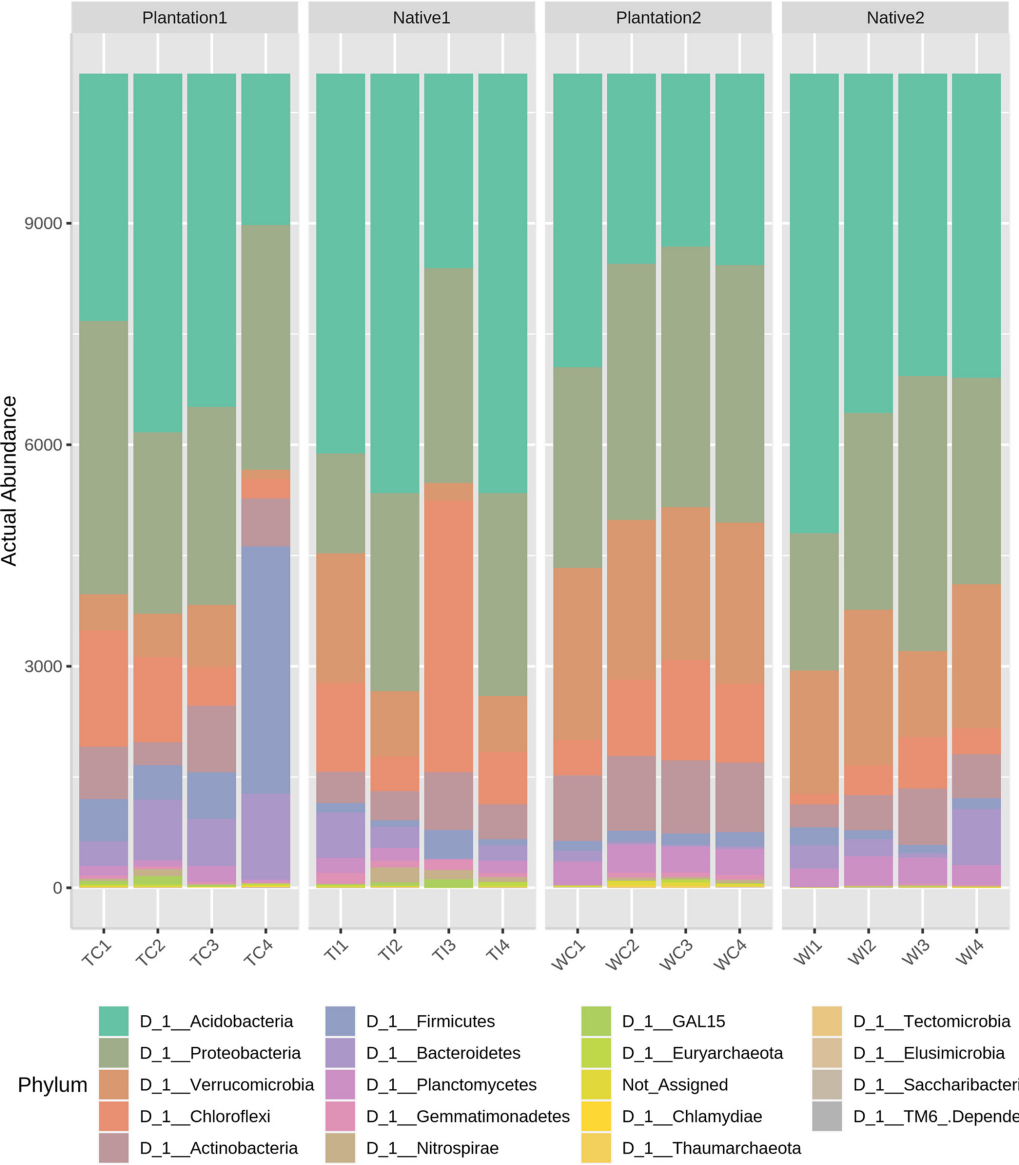
Relative abundance plot of the microbial communities in the native and temperate pine forests.

Land use change influenced the bacterial communities, as the microbiome in the native forests differed from those in the temperate pine plantations. For abundance profiling, OTU data were summarized, and their abundance was compared at the phylum level based on the annotation. The overall abundance of OTUs was visualized using a stacked bar plot as shown in Fig. 1. *Acidobacteria* (17.89% to 54.74%) was the most abundant phylum, and *Proteobacteria* (16.74% to 30.96%) and *Verrucomicrobia* (0.99% to 14.53%) were next. Across all sites, the temperate pine forests constantly influenced the acidobacterial communities which were relatively higher in the native forests.

### Data availability.

The 16S rRNA gene amplicon raw read data set is available at the NCBI Sequence Read Archive (SRA) under BioProject PRJNA715835 with accession numbers SRR14023269 (TC1), SRR14023268 (TC2), SRR14023277 (TC3), SRR14023276 (TC4), SRR14023275 (TI1), SRR14023274 (TI2), SRR14023273 (TI3), SRR14023272 (TI4), SRR14023271 (WC1), SRR14023270 (WC2), SRR14023267 (WC3), SRR14023266 (WC4), SRR14023265 (WI1), SRR14023280 (WI2), SRR14023279 (WI3), and SRR14023278 (WI4).
